# Managing the Giant: A Novel Approach in Orthotopic Heart Transplantation With Extreme Left Atrial Enlargement

**DOI:** 10.1002/ccr3.70655

**Published:** 2025-07-27

**Authors:** Lorenzo Giovannico, Federica Mazzone, Giuseppe Fischetti, Domenico Parigino, Luca Savino, Tommaso Acquaviva, Aldo Domenico Milano, Tomaso Bottio

**Affiliations:** ^1^ Cardiac Surgery Unit, Department of Precision and Regenerative Medicine and Ionian Area (DiMePRe‐J) University of Bari Medical School Bari Italy

**Keywords:** atrial reconstruction, bovine pericardial patch, giant left atrium, orthotopic heart transplantation, superior vena cava syndrome

## Abstract

This case report highlights an innovative surgical approach in orthotopic heart transplantation (OHT) for a patient with the largest recorded giant left atrium, measuring a biplanar volume of 1906 mL. The patient, a 63‐year‐old man with severe heart failure and a history of mitral and aortic valve replacements, presented with atrial fibrillation, moderate systolic dysfunction, and severe cardiomegaly. Despite optimized medical therapy, his condition warranted heart transplantation. The procedure involved a novel surgical technique utilizing Teflon reinforcement and a bovine pericardial patch to reconstruct the significantly enlarged left atrium. Postoperative outcomes were favorable, with no rejection episodes and resolution of pulmonary symptoms. This report underscores the challenges of atrial size mismatch in OHT and the necessity for tailored surgical strategies to address extreme anatomical variations. The successful outcome exemplifies the potential of customized approaches in complex heart transplant cases.

**Abbreviations**
CK‐MB: creatine phosphokinase‐MB
GLA: giant left atrium
hs‐TnI: high sensitivity troponin I
LVEF: left ventricle ejection fraction
OHT: orthotopic heart transplantation
PAPm: mean pulmonary artery pressure
PAPs: systolic pulmonary artery pressure
PCWP: pulmonary capillary wedge pressure
PVR: pulmonary vascular resistance
TAPSE: tricuspid annular plane systolic excursion

**Summary**

- This case highlights a novel surgical approach in orthotopic heart transplantation for extreme left atrial enlargement.
- Tailored atrial reconstruction using Teflon reinforcement and a bovine pericardial patch allowed successful transplantation, demonstrating the importance of customized techniques in addressing severe anatomical challenges in cardiac surgery.

## 1 Introduction

Giant left atrium (GLA) is defined as an anteroposterior diameter exceeding 8 cm [1], classically associated with rheumatic mitral valve regurgitation, with a reported incidence of 0.3% [2, 3]. This case details a novel technique successfully employed in a patient with the largest left atrium ever reported in a heart transplantation recipient.

## 2 Case History

**FIGURE 1:**
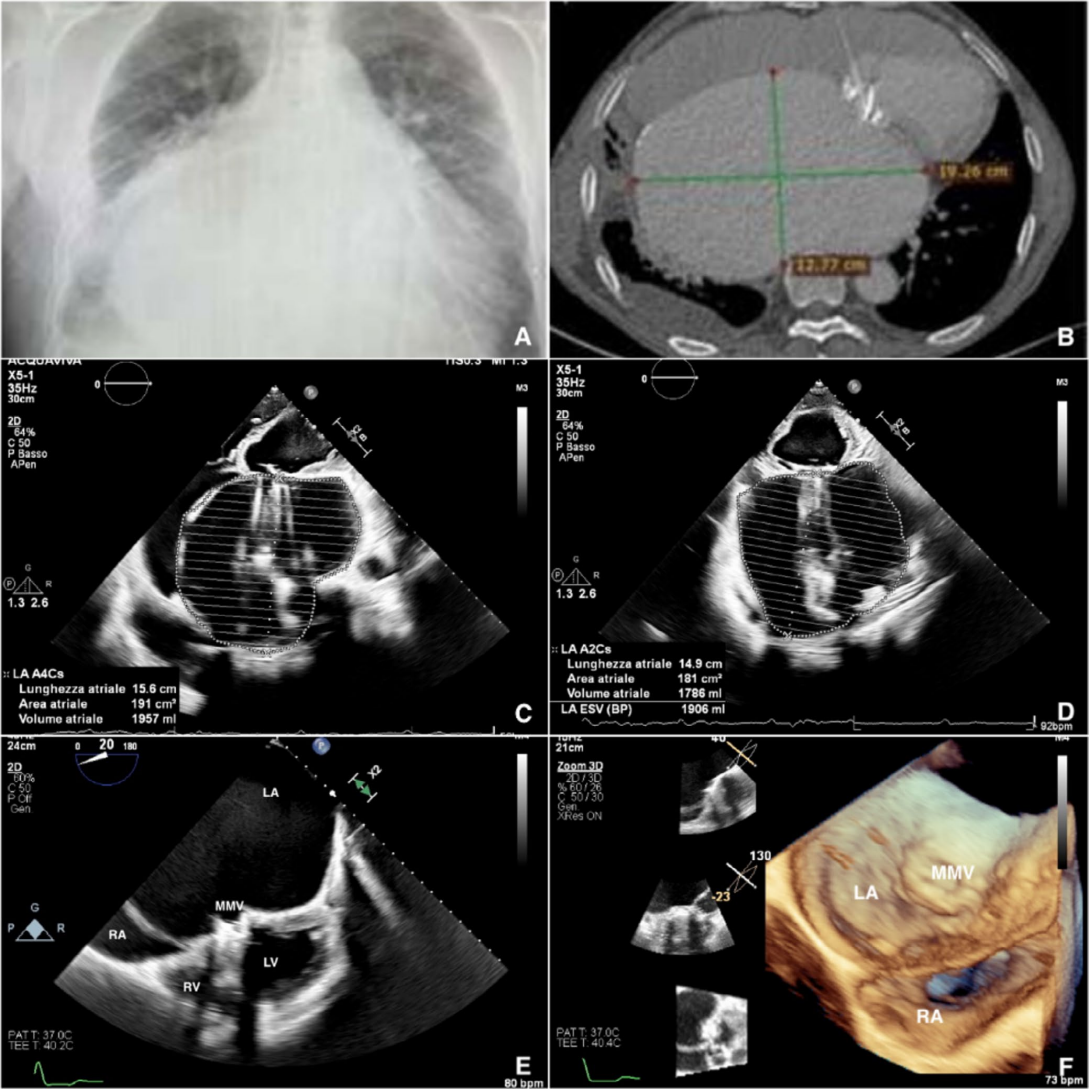
Pre‐operative imaging: Chest X ray (A); CT scan in coronal view (B); Transthoracic echocardiogram with end‐systolic LA biplane volume (C, D); Transesophageal echocardiogram 2D mid esophageal 4‐chamber view (E) and 3D real time view (F). LA, left atrium; LV, left ventricle; MMV, mechanical mitral valve; RA, right atrium; RV, right ventricle.

A 63‐year‐old man presented to our Heart Failure and Transplant Unit 36 years after receiving a mitral and aortic valve replacement with 29‐mm and 23‐mm Omnicarbon mechanical prostheses (Medical Inc., Inver Grove Heights, MN), respectively, for rheumatic heart disease. In 2018, he underwent reoperation for aortic valve stenosis, receiving a 23‐mm Omnicarbon replacement; however, due to prohibitive surgical risk, the dysfunctional mitral prosthesis was left in situ. In the preceding months, he experienced two hospitalizations for dyspnea and congestive heart failure. At presentation, he exhibited congestive heart failure, dyspnea with minimal exertion, pitting edema, and dysphagia. His medical regimen included optimized therapy with sacubitril/valsartan, beta‐blocker, aldosterone antagonist, SGLT2 inhibitor, and 125 mg daily of furosemide. Electrocardiography showed atrial fibrillation with a mean ventricular rate of 70 bpm. Chest X‐ray (Figure 1A) revealed severe cardiomegaly. Transthoracic (Figure 1C,D) and transesophageal (Figure 1E,F) echocardiography confirmed a GLA (biplanar volume 1906 mL, volume/BSA 1013 mL/m^2^, area 191 cm^2^), along with moderate mitral prosthesis stenosis (mean gradient 10 mmHg) and severe insufficiency due to intra‐ and paraprosthetic regurgitation. Both ventricles demonstrated moderate systolic dysfunction (left ventricle ejection fraction [LVEF] 42%, tricuspid annular plane systolic excursion [TAPSE] 15 mm), accompanied by severe tricuspid regurgitation and hepatic congestion. Computed tomography (CT) scan (Figure 1B) confirmed the left atrial dimensions (laterolateral diameter 19.26 cm, anteroposterior diameter 12.77 cm) and showed compression of adjacent cardiac structures and lung parenchyma, with a left pleural effusion. Performing a magnetic resonance imaging (MRI) on the patient was not possible because the mechanical mitral prosthesis was incompatible with this diagnostic procedure.

## 3 Differential Diagnosis

- Severe cardiomegaly secondary to long‐standing mitral valve disease;
- Heart failure due to atrial fibrillation‐induced cardiomyopathy;
- Pulmonary hypertension due to left atrial enlargement;
- Prosthetic mitral valve dysfunction;

## 4 Conclusion and Results

**FIGURE 2:**
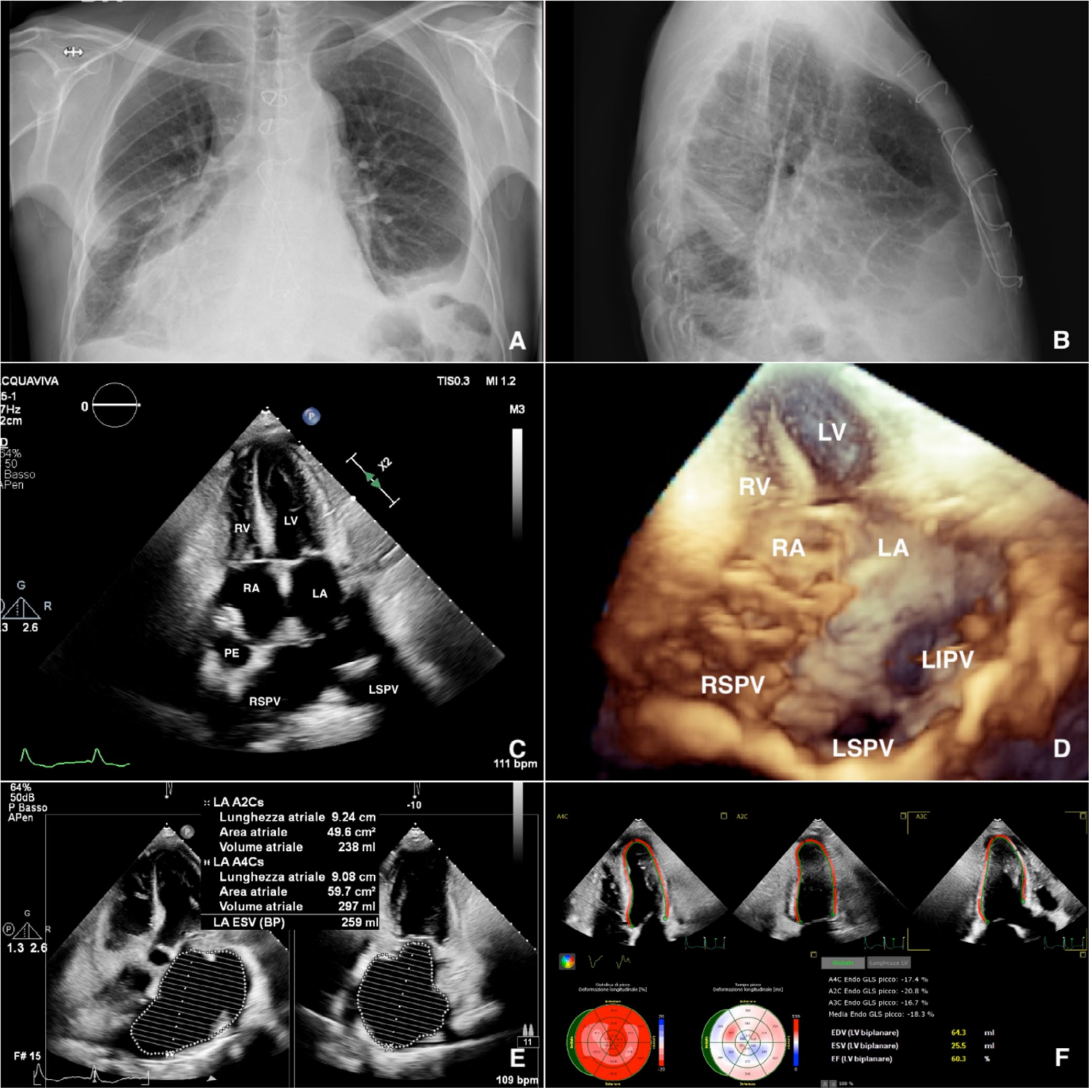
Post‐operative imaging: Chest X ray (A, B); Transthoracic echocardiogram 2D apical 4‐chamber (C) and real time 3D (D), biplane end‐systolic LA volume (E), global longitudinal strain of −18% and biplane left ventricular ejection fraction of 60% (F). LA, left atrium; LIPV, left inferior pulmonary vein; LSPV, left superior pulmonary vein; LV, left ventricle; PE, pericardial effusion; RA, right atrium; RSPV, right superior pulmonary vein; RV, right ventricle.

We proceeded to screen the patient for eligibility for the heart transplant waiting list. The right heart catheterization indicated moderate combined pulmonary hypertension (PAPs 72 mmHg, PAPm 42 mmHg, PCWP 24 mmHg, PVR 2.5 WU), along with a reduced cardiac index (2 L/min/m^2^) and cardiac output (3.9 L/min). After 2 weeks of treatment with dobutamine infusion therapy at 5 μg/kg/min and furosemide at 250 mg/day, repeat right heart catheterization showed reductions in pulmonary artery pressures (PAPs 50 mmHg, PAPm 29 mmHg, PAWP 18 mmHg, PVR 1.9 WU). Consequently, the patient was listed for heart transplantation. A suitable donor, a 40‐year‐old male who succumbed to a cerebral hemorrhage, became available 25 days later. Cardiac markers (hs‐TnI, CK‐MB) were within normal limits, indicating no evidence of myocardial injury. Transthoracic echocardiography revealed normal biventricular function (LVEF 60%, TAPSE 24 mm) without significant valvular disease. Coronary angiography demonstrated no critical stenosis, and a negative virtual crossmatch was obtained. We performed a cardiectomy followed by an orthotopic heart transplant using the standard bicaval technique. The significantly enlarged left atrium was reinforced with an external Teflon strip and augmented with a large St. Jude Medical bovine pericardial patch (Abbott Laboratories, Chicago, IL, USA) to accommodate the new graft. The inferior vena cava and pulmonary artery were directly anastomosed. A 30‐mm Gelweave vascular graft (Terumo Aortic, Scotland, UK), reinforced with an external Teflon strip, was interposed in the ascending aorta, and the superior vena cava was anastomosed using a St. Jude Medical bovine pericardial patch (Abbott Laboratories, Chicago, IL, USA). The operation proceeded without significant complications; ischemic time was 2 h and 55 min. The cytotoxic crossmatch was negative, and the patient was extubated several hours post‐transplant. The postoperative course was complicated by superior vena cava syndrome (SVCS), presenting as upper extremity edema, attributed to perianastomotic inflammation causing anastomosis stenosis. This resolved spontaneously within 10 days. Due to thromboembolic risk associated with the reconstructed left atrium, the patient received warfarin anticoagulation, targeting an international normalized ratio of 2–3. Two postoperative endomyocardial biopsies revealed no evidence of acute cellular rejection (ISHLT 04 0R [4]). At discharge, 25 days post‐transplant, chest radiography (Figure 2A,B) showed no pulmonary consolidations, and transthoracic echocardiography demonstrated a left atrium biplanar volume of 259 mL (Figure 2C–E) and good biventricular function (Figure 2F). Six months post‐transplant, the patient had returned to work and resumed normal activities.

## 5 Discussion

Significant atrial size mismatch is a frequent challenge in orthotopic heart transplantation (OHT), often arising from the cardiomegaly and biatrial enlargement common in end‐stage cardiomyopathy secondary to chronic mitral and/or tricuspid valvular disease. While atrial plication, as described by Duncan et al. [5], is a well‐established technique to address this mismatch by reducing recipient atrial size, its efficacy is limited by the degree of enlargement. Successful plication has been reported in cases with moderately enlarged atria (volume 350 mL) [6, 7], but more extensive surgical approaches may be necessary for patients with significantly larger atria. Bishay et al. [8] described a case of GLA (84 cm^2^) requiring complete resection, preserving only the pulmonary venous ostia, followed by extensive pulmonary vein mobilization to facilitate anastomosis. Soquet et al. [9] presented an alternative approach for a particularly large GLA (113 cm^2^, 698 mL/m^2^), creating a pulmonary venous confluence using a Gore‐Tex patch, inspired by total anomalous pulmonary venous return. However, even this innovative approach proved insufficient for our patient's exceptionally large GLA, necessitating further modifications to the surgical technique. Specifically, vascular prostheses were necessary for ascending aortic anastomosis, and a bovine pericardial patch was used for lengthening and allowing the superior vena cava anastomosis. The markedly enlarged left atrial volume (1906 mL), in conjunction with evidence of moderate biventricular dysfunction—documented via transthoracic echocardiography (left ventricular ejection fraction 40%, TAPSE 15 mm) and confirmed by invasive hemodynamic assessment (cardiac index 2.0 L/min/m^2^) prompted consideration of orthotopic heart transplantation as the most appropriate therapeutic strategy.

Postoperatively, anticoagulation with warfarin was initiated to mitigate the heightened risk of thromboembolism associated with the reconstructed atrium using a bovine pericardial patch and Teflon reinforcement. Additional contributing factors included the patient's history of atrial fibrillation and residual atrial enlargement, both known to favor blood stasis and thrombus formation.

In addition to thromboembolic concerns, the use of bovine pericardium carries intrinsic risks that must be considered. Literature from congenital heart surgery, where bovine pericardial patches are frequently used for intracardiac repairs and vascular reconstruction, reports complications such as patch infection, calcification, pseudoaneurysm formation, and, rarely, rupture. These adverse events often occur in long‐term follow‐up, particularly when the patch is exposed to systemic pressures or high‐flow environments. Although our patient experienced no such issues in the early postoperative period, careful monitoring is essential. In congenital repairs, studies such as those by Elassal et al. and Peivandi et al. highlight these complications, reinforcing the need for vigilance even in adult transplant settings where bovine pericardium is used in non‐standard anatomical reconstructions [10, 11].

In our case, the postoperative course was complicated by transient SVCS, manifested as upper extremity edema. This was attributed to perianastomotic inflammation leading to localized stenosis at the superior vena cava anastomosis site. While SVCS has not been frequently reported with traditional left atrial plication or partial resection techniques, the extensive atrial reconstruction with bovine pericardial patch and Teflon reinforcement, combined with vascular prostheses use, likely increased the risk of anastomotic tension and inflammation. The condition resolved spontaneously without the need for surgical revision. This highlights that complex reconstruction techniques, while necessary in extreme anatomical cases, may pose unique postoperative risks compared to standard size‐mismatch correction strategies [12, 13, 14].

Superior vena cava syndrome is a rare but potentially serious complication following orthotopic heart transplantation. It can occur early in the postoperative period, as illustrated by the case reported by De Haes et al., in which a patient developed upper body cyanosis and edema due to extrinsic compression of the superior vena cava by surgical materials and clots. This was promptly diagnosed using transesophageal echocardiography and resolved through immediate bedside surgical intervention [15].

A broader retrospective study by Aronowitz et al. identified complex superior venous anatomy—such as prior cavopulmonary connections or congenital anomalies—as a significant risk factor for SVC obstruction requiring reintervention. Specifically, complex reconstructive strategies, including the use of patches or donor innominate veins, were associated with a markedly higher rate of reintervention compared to direct SVC‐to‐SVC anastomosis or interposition grafts [16].

These findings underscore the importance of individualized surgical planning in patients with abnormal venous anatomy and highlight the critical role of early postoperative imaging in the prompt identification and management of SVCS.

To the best of our knowledge, this represents the first reported case of OHT involving such a large GLA, employing this innovative surgical technique.

## 6 Conclusion

This case highlights the feasibility of a novel atrial reconstruction technique in OHT for extreme GLA. Tailored surgical approaches are crucial for successful transplantation in cases of significant anatomical variations.

## Author Contributions

**Lorenzo Giovannico:** conceptualization, writing – original draft, writing – review and editing. **Federica Mazzone:** supervision. **Giuseppe Fischetti:** conceptualization, validation, visualization. **Domenico Parigino:** methodology, writing – original draft. **Luca Savino:** data curation. **Tommaso Acquaviva:** visualization. **Aldo Domenico Milano:** conceptualization, supervision, writing – review and editing. **Tomaso Bottio:** data curation, formal analysis, supervision, writing – review and editing.

## Consent

The authors should confirm that written informed consent was obtained from the patient to publish this report in accordance with the journal's patient consent policy.

## Conflicts of Interest

The authors declare no conflicts of interest.

## Data Availability

The data supporting the findings of this study are available from the corresponding author [LG] upon reasonable request. The data are not publicly available due to privacy restrictions.
